# Hypoxia-inducible factor-1α promotes cell survival during ammonia stress response in ovarian cancer stem-like cells

**DOI:** 10.18632/oncotarget.23010

**Published:** 2017-12-07

**Authors:** Shojiro Kitajima, Kian Leong Lee, Hiroki Hikasa, Wendi Sun, Ruby Yun-Ju Huang, Henry Yang, Shinji Matsunaga, Takehiro Yamaguchi, Marito Araki, Hiroyuki Kato, Lorenz Poellinger

**Affiliations:** ^1^ Cancer Science Institute of Singapore, National University of Singapore, Singapore, Singapore; ^2^ Pharmacology, Graduate School of Medicine, Osaka City University, Osaka, Japan; ^3^ Cancer and Stem Cell Biology Program, Duke-NUS Medical School, Singapore, Singapore; ^4^ Department of Biochemistry, School of Medicine, The University of Occupational and Environmental Health, Kitakyushu, Japan; ^5^ School of Biological Sciences, Nanyang Technological University, Singapore, Singapore; ^6^ Department of Transfusion Medicine and Stem Cell Regulation, Juntendo University Graduate School of Medicine, Tokyo, Japan; ^7^ Department of Cell and Molecular Biology, Karolinska Institute, Stockholm, Sweden

**Keywords:** hypoxia-inducible factors, ammonia, glutamine synthetase, energy metabolism, cancer stem cells

## Abstract

Ammonia is a toxic by-product of metabolism that causes cellular stresses. Although a number of proteins are involved in adaptive stress response, specific factors that counteract ammonia-induced cellular stress and regulate cell metabolism to survive against its toxicity have yet to be identified. We demonstrated that the hypoxia-inducible factor-1α (HIF-1α) is stabilized and activated by ammonia stress. HIF-1α activated by ammonium chloride compromises ammonia-induced apoptosis. Furthermore, we identified glutamine synthetase (GS) as a key driver of cancer cell proliferation under ammonia stress and glutamine-dependent metabolism in ovarian cancer stem-like cells expressing CD90. Interestingly, activated HIF-1α counteracts glutamine synthetase function in glutamine metabolism by facilitating glycolysis and elevating glucose dependency. Our studies reveal the hitherto unknown functions of HIF-1α in a biphasic ammonia stress management in the cancer stem-like cells where GS facilitates cell proliferation and HIF-1α contributes to the metabolic remodeling in energy fuel usage resulting in attenuated proliferation but conversely promoting cell survival.

## INTRODUCTION

Ammonia is a toxic by-product of mammalian metabolism that is liberated from amino acids mainly through glutaminolysis (a series of catabolic reactions of glutamine) [1, 2]. Although it has been demonstrated that cancer cells rely more on glutamine for their rapid and continuous proliferation compared to intact cells [3–5], it remains unclear what are the signal transduction pathways activated by ammonia, which can be essential for sensing overabundance of the by-product, adaptation towards cellular stresses and subsequent tumor growth. Toxic effects of ammonia are known, by a number of articles, to inhibit cell growth [6, 7] or induce cell death [8–10]. Interestingly, in those studies, the severity of the effects caused by ammonia is widely variable, suggesting unknown mechanisms with key roles in stress response under conditions of high ammonia, and thus in glutamine-dependent cancer progression.

The present study was designed to demonstrate an involvement of the hypoxia-inducible factors (HIFs) in stress response pathways against ammonia. HIFs were identified as oxygen-regulated transcriptional activators [11, 12]. Under normal oxygen conditions (normoxia), HIF proteins are hydroxylated by a family of O_2_-dependent prolyl hydroxylase domain-containing enzymes (PHD) and constitutively degraded by the ubiquitin-proteasome system [13–16]. PHD activity is inhibited under conditions of low oxygen (hypoxia) as well as by cobalt chloride (CoCl_2_) or iron-chelators. Therefore, the PHD-HIF axis is the core signaling machinery that mediates cellular responses towards hypoxia via transcriptional regulation. It is known that HIFs are also activated under normoxia by reactive oxygen species (ROS) [17], nitric oxide (NO) [18, 19] or mutations of a mitochondrial enzyme [20]. These findings have revealed additional HIF functions for diverse metabolic stress responses. However, the understanding of the role of HIFs in stress response remains limited.

Here, we identify that HIF-1α is activated in response to high ammonia. This compromises cell growth by modifying cellular energy metabolism that leads to promoted survival against ammonia-induced cell death. We also found that HIF-1α activation levels are elevated by the depletion of glutamine synthetase (GS) that integrates regulations of energy metabolism and cellular stress response. For ammonia disposal in peripheral tissue, GS is a key limiting enzyme that catalyzes the condensation of ammonia and glutamate to glutamine. Importantly, recent studies implicated GS in tumor-promoting enzymes that enhance nucleotide biosynthesis [21, 22]. In the present study, we examined the detailed roles of HIF-1α and GS in mechanisms of the ammonia-derived cellular stress response.

## RESULTS

### Stress tolerance to ammonia is important for cancer stem-like cells

**Figure 1:**
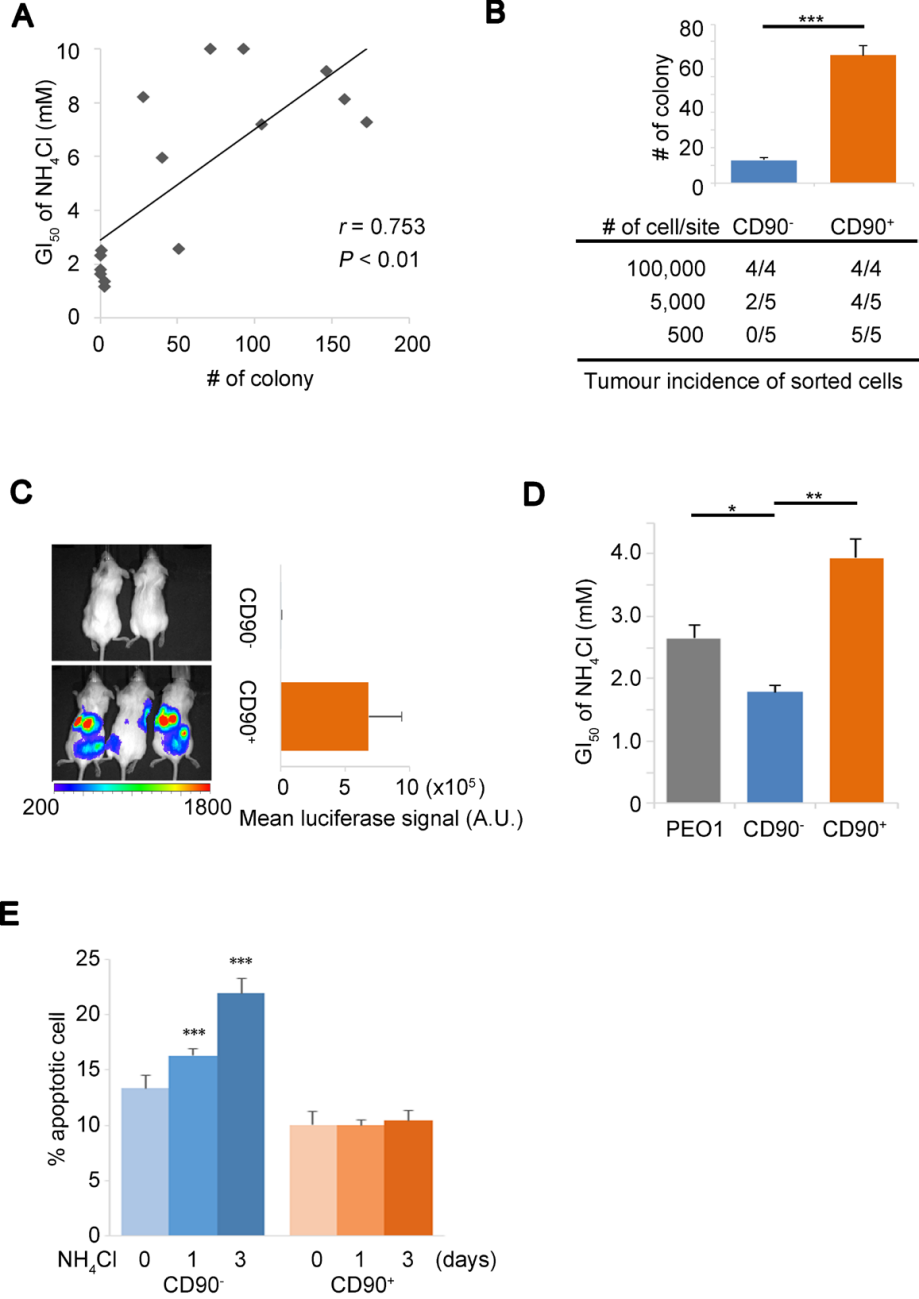
Ammonia tolerance enhances putative tumor initiation (**A**) Correlation plot of the GI_50_ of NH_4_Cl versus the number of colonies in soft agar for 15 OVC cell lines. r; Pearson correlation coefficient. (**B**) CD90^+^ and CD90^−^ subpopulations of PEO1 cells were isolated by cell-sorting and established. Characteristics of cells were assessed *in vitro* by soft agar assays with an initial seeding number of 2,000 (bar chart, upper panel) and *in vivo* by serial dilution xenograft assays (table, lower panel) that show the incidence of tumors out of the total number of injection sites. (**C**) *In vivo* orthotopic xenograft of CD90^−^ (2 mice) or CD90^+^ (3 mice) PEO1 cells infected with a luciferase-expressing vector. 2 × 10^6^ cells were injected into the peritoneal cavity. The signals from the engrafted cells were detected with IVIS imaging system 4 weeks after injection (Left). A.U., arbitrary units. Right panel shows average intensities of luciferase bioluminescence. (**D**) Determination of the GI_50_ of NH_4_Cl for the parental cell line (PEO1), CD90^−^ and CD90^+^ subpopulations. (**E**) Apoptotic and live cell population were determined using Annexin-V-Alexa 647 and Sytox-blue staining in CD90^−^ and CD90^+^ cells after 10 mM NH_4_Cl treatment for the indicated time. Error bars indicate s.e.m. ^*^P < 0.05; ^**^P < 0.01; ^***^P < 0.001 (Student’s *t*-test).

Although a number of studies have reported that ammonia has inhibitory effects on cell proliferation [6, 7, 9], the regulatory mechanisms of cellular response and their implications in tumor growth have not been widely studied. We first analyzed the cellular growth data of a previous study [6] and found an inverse relationship between the ammonia levels in culture media and the 50% growth inhibition (GI_50_) of NH_4_Cl (*r* = −0.61, Supplementary Figure 1A). To independently validate these findings, we tested the tolerance of 15 ovarian cancer (OVC) cell lines with ammonia gradients to determine their NH_4_Cl GI_50_. We consistently found a strong and significant correlation between the GI_50_ of NH_4_Cl and the colony forming capacity of the OVC cells in soft agar that represented anchorage-independent growth advantage (Figure 1A and Supplementary Table 1). These findings raised the question as to what mechanisms underlie and what defines the tolerance to ammonia and moreover the continued cell proliferation. To figure out the detailed mechanisms by which cancer cells respond to ammonia, we established a cell-based platform. We isolated a CD90-positive (CD90^+^) cell subpopulation from PEO1 ovarian cancer cell line [23, 24] (Supplementary Figure 1B), which had cancer stem-like properties including a high capacity of stress tolerance. CD90^+^ PEO1 cells generated the greater number of colonies in soft agar and had the increased rates of tumor incidence in serial dilution xenograft assays compared to CD90^−^ cells (Figure 1B, Supplementary Figure 1C). Consistently, CD90^+^ cells also showed dramatically better tumor growth upon intraperitoneal (i.p.) injection than CD90^−^ cells (Figure 1C). These data demonstrate that CD90^+^ PEO1 cells have high tumorigenicity, which can be relevant for CSCs, although the value of CD90 antigen as a CSC marker remains controversial. Importantly, the GI_50_ of NH_4_Cl of CD90^+^ PEO1 cells was significantly higher than that of CD90^−^ cells (Figure 1C), suggesting a link between the tolerance to ammonia and tumor growth. In addition, while the rates of ammonia-induced apoptosis were clearly increased in CD90^−^ cells, no significant change was seen in CD90^+^ cells upon 10 mM NH_4_Cl treatment up to 3 days (Figure 1E). These findings collectively suggest that tolerating cellular stresses posed by ammonia is an important property for tumorigenesis and subsequent tumor growth and that CD90^+^ PEO1 cells have CSC-like properties and are tolerant to ammonia stress. Hence, the CD90^+^/CD90^−^ PEO1 system has been demonstrated to be suitable for following studies.

### HIF-α proteins are activated during stress response to ammonia

**Figure 2:**
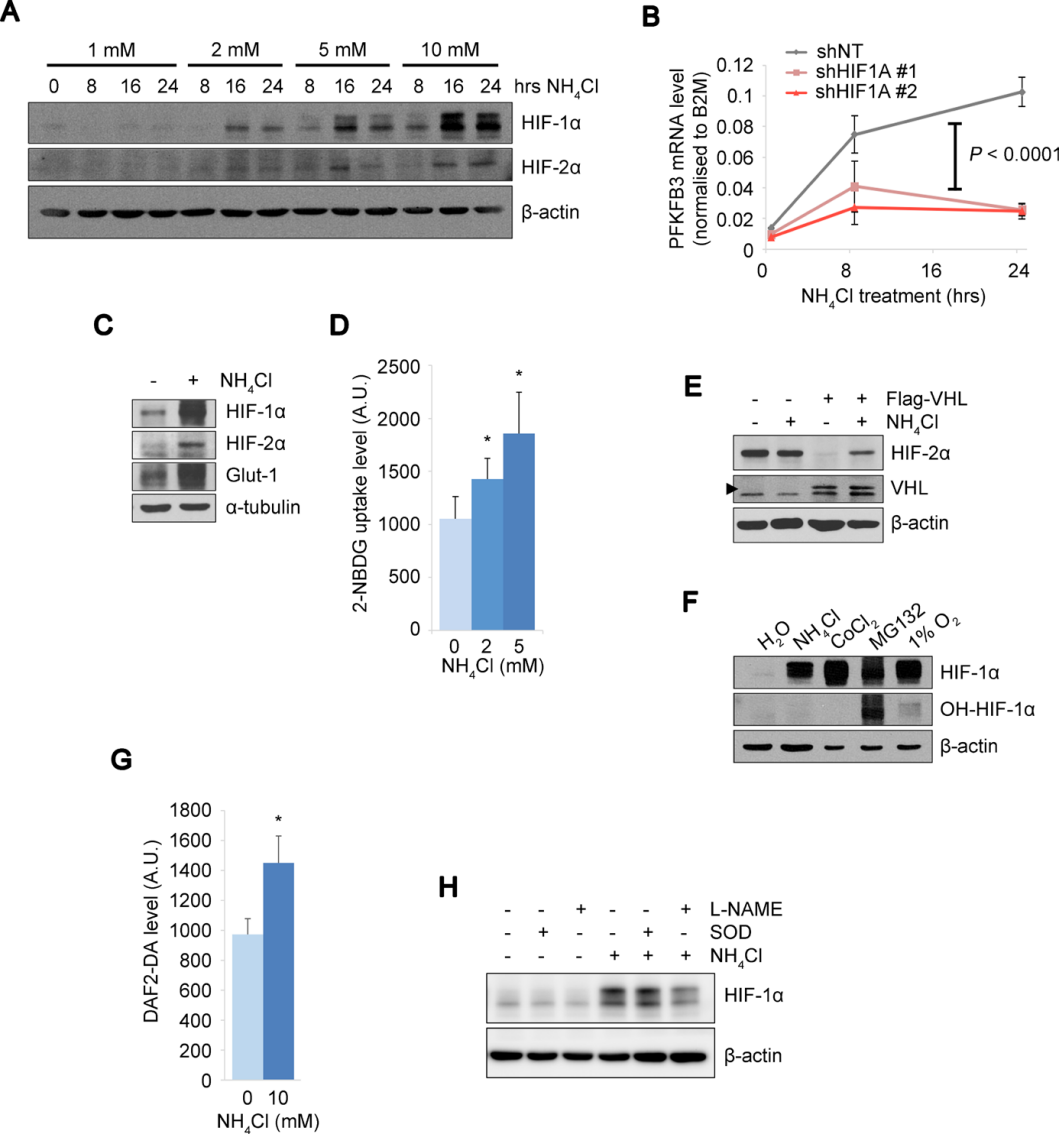
The PHD-HIF axis up-regulates glycolysis under ammonia stress (**A**) HIF-1α and HIF-2α levels were increased by NH_4_Cl treatment in a dose- and time-dependent manner. SKOV3 cells treated with NH_4_Cl at the indicated concentrations and durations. β-actin expression was shown as the loading control. (**B**) Kinetic PFKFB3 mRNA expressions in PEO1 CD90^+^ cells upon NH_4_Cl treatment. The PFKFB3 up-regulation by NH_4_Cl observed in control cells (shNT) was almost completely abolished by two independent shRNA knockdown of HIF1A (HIF-1α). Each duplicated experiment was repeated twice. A two-way ANOVA and post-hoc tukey comparisons revealed a group effect between NT and KDs (*P* < 0.0001). (**C**) Protein expressions of HIFs, a HIF target GLUT1 and α-tubulin as a loading control were determined in SKOV3 cells by immunoblot upon NH_4_Cl treatment (10 mM) for 16 hrs. (**D**) Glucose uptake assay with NH_4_Cl treatment at 0, 2 or 5 mM for 16 hours in CD90^−^ and CD90^+^ PEO1 cells. Intracellular glucose levels were determined using 2-NBDG fluorescence signals. (**E**) Immunoblot of A498 cells and their derivative cells expressing WT-VHL upon treatment with 10 mM NH_4_Cl. Arrow head indicates the ectopic expression of WT-VHL. (**F**) Comparison of prolyl-hydroxylation levels of HIF-1α between the indicated treatments. SKOV3 cells were treated with NH_4_Cl (10 mM for 16 hrs), CoCl_2_ (150 μM for 1 hr), MG132 (10 μM for 2 hrs) or hypoxia (1% O_2_ for 2 hrs). The amount of protein loaded was adjusted to show approximately the same level of whole HIF-1α protein (refer to β-actin levels). (**G**) Cellular NO levels were determined by the fluorescent signal from DAF2-DA dye. (**H**) Effect of L-NAME or SOD on HIF-1α expression levels by NH_4_Cl. The data in D and G show the mean of at least *n* = 3 independent experiments. Error bars indicate s.e.m. ^*^P < 0.05; (Student’s *t*-test).

On the basis of the results implicating the tolerance to ammonia in a property of CD90^+^ PEO1 CSC-like cells, we attempted to identify cellular mediators of their stress response. We found that HIF-1α and –2α proteins were accumulated upon NH_4_Cl treatment in a dose- and time-dependent manner (Figure 2A). To test whether the HIF expression had physiological activities under conditions of high ammonia concentration, we next determined mRNA level of *PFKFB3* and protein level of GLUT-1, canonical HIF target glycolytic factor and glucose transporter, respectively. The mRNA expression of *PFKFB3* was elevated in a time-dependent manner by NH_4_Cl treatment and this became largely abolished by two independent HIF-1α knockdowns (Figure 2B). Importantly, the GLUT-1 protein expression was elevated and likewise, the glucose uptake was significantly increased under ammonia stress conditions with NH_4_Cl treatment (Figure 2C, 2D). These results suggested that glycolysis is up-regulated in response to ammonia via HIF pathway activation, which is consistent with the physiological role of HIFs under hypoxia. To investigate the mechanisms underlying the activation of HIFs by ammonia, we next examined the HIF degradation machinery, which is essential for the stabilization of the HIFs when disabled typically under hypoxic conditions. We employed A498, a renal cell carcinoma (RCC) cell line, which is defective for the von Hippel-Lindau (VHL) factor and constitutively expresses HIF-2α as well as an A498 derivative cell line expressing exogenous wild-type (WT) VHL. NH_4_Cl treatment did not alter HIF-2α expression levels in the parental VHL-mutated A498 cells, while HIF activation by NH_4_Cl was restored in the presence of WT-VHL (Figure 2E). This suggests that ammonia affects VHL-mediated protein degradation of the HIFs [13, 25]. To further investigate the targets of ammonia that is involved in HIF degradation, we assessed the level of prolyl-hydroxylation of HIF-1α (OH-HIF) that is required for the subsequent ubiquitin-mediated degradation of HIF proteins [12, 14]. Interestingly, HIF-1α proteins were accumulated without significant changes in the hydroxylation status upon NH_4_Cl, cobalt chloride (CoCl_2_) or hypoxia treatment while treatment with a proteasome inhibitor MG132 resulted in high HIF-1α expression with substantial hydroxylation level (Figure 2F). This suggests that similarly to CoCl_2_ or hypoxia, ammonia may directly or indirectly blunt PHD activity by unidentified mechanisms and thus lead to the HIF protein stabilization. It is important to note that consistent with previous studies [26, 27], we observed elevated levels of cellular nitric oxide (NO) upon NH_4_Cl treatment (Figure 2G), which might lead to PHD inhibition [28]. The NO production we observed could be catalyzed by NOS that consumes arginine produced from ammonia and mediated by urea cycle-related enzymes. Thus we next tested the effect of a NOS inhibitor, N(G)-Nitro-L-arginine methyl ester (L-NAME), on HIF-1α expression. As we expected, L-NAME treatment attenuated HIF-1α up-regulation by NH_4_Cl while Superoxide dismutase (SOD), a quencher of another species of reactive oxygen, did not affect HIF-1α levels (Figure 2H). Collectively, we demonstrate that HIF proteins are stabilized and activated by ammonia, which may inhibit PHDs through NOS-mediated NO production that can lead to the disrupted HIF degradation. Of note, this in turn up-regulates glucose uptake and glycolysis pathways with important implications for changes in cancer cell metabolism.

### HIF-1α suppresses cell growth but promotes survival against ammonia-induced apoptosis

**Figure 3:**
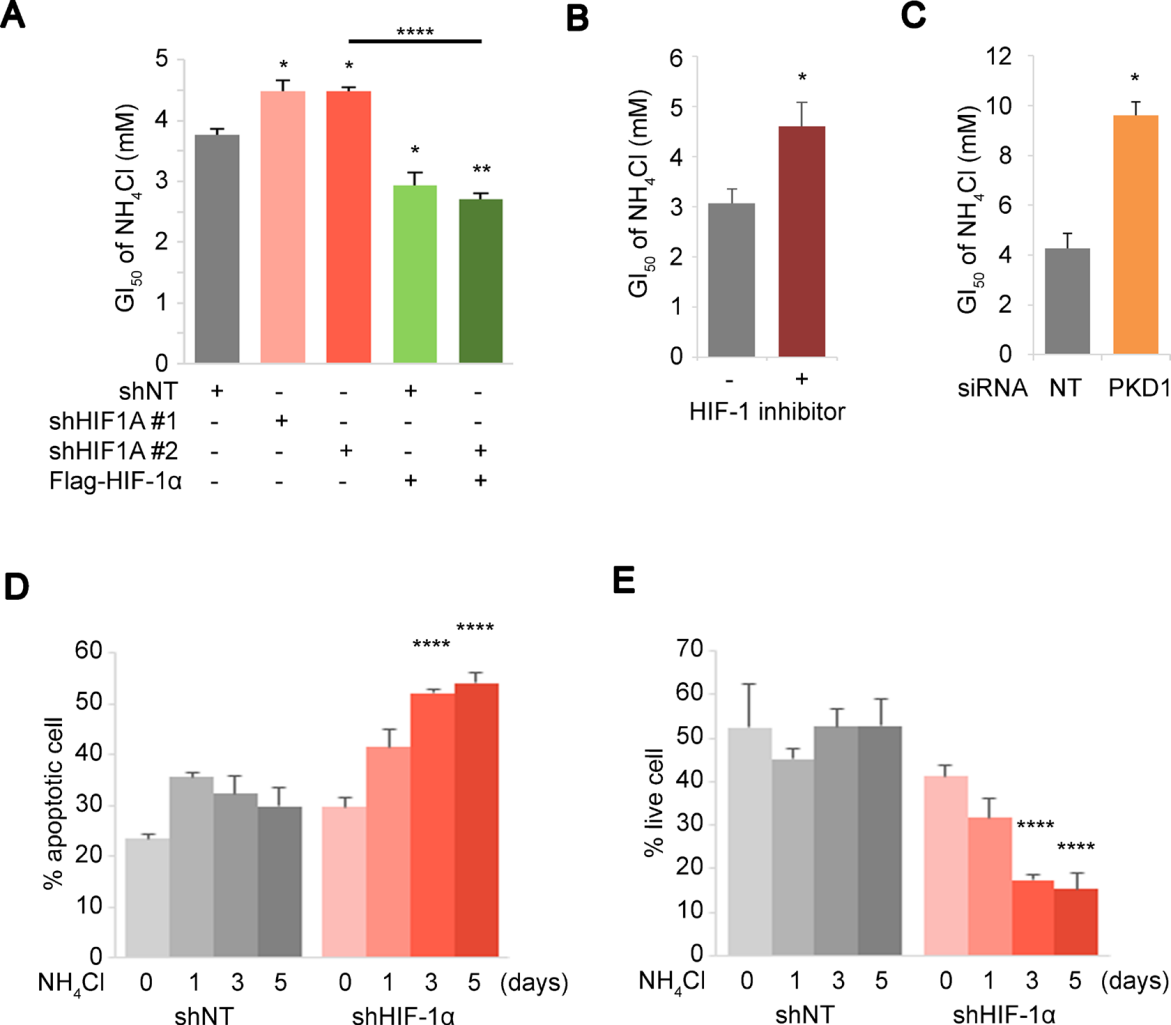
HIF expression level mediates cellular response towards survival under ammonia stress (**A**–**C**) Inhibitions of the HIF pathway results in improved ammonia tolerance. (A) Changes in the GI_50_ of NH_4_Cl were determined during HIF-1α depletion by shRNA and/or Flag-HIF-1α rescue overexpression. (B, C) Effect of 30 μM of a HIF-1α inhibitor 400083 (B) or PDK1 siRNA (C) on the GI_50_ of NH_4_Cl was tested in PEO1^+^ cells. (**D, E**) Apoptotic and live cell population were determined by Annexin-V assays upon 10 mM NH_4_Cl treatment for the indicated time. CD90^+^ PEO1 cells were used in (A–E) which show the mean of *n* = 3 independent experiments. Error bars indicate s.e.m. ^*^P < 0.05; ^**^P < 0.01; ^****^P<0.0001 (Student’s *t*-test).

To elucidate the biological implications of HIF activations for stress response to ammonia, we determined the NH_4_Cl GI_50_ in the CD90^+^ PEO1 cells in the presence or absence of HIF-1α knockdown (HIF-1α KD) and/or Flag-HIF-1α overexpression. Two independent knockdowns of HIF-1α increased the GI_50_ of NH_4_Cl in CD90^+^ PEO1 cells while overexpression of Flag-HIF-1α decreased the GI_50_ significantly (Figure 3A lane 1,2,3 and 4). The ectopic expression of Flag-HIF-1α over shHIF-1α resulted in the significant expression level of HIF-1α (Supplementary Figure 2A) and the lower GI_50_ compared to the knockdown alone (Figure 3A lane 5). Consistent with HIF-1α depletion, a HIF-1α inhibitor compound 400083 and knockdown of the glycolytic enzyme PDK1 also increased the GI_50_ of NH_4_Cl (Figure 3B, 3C and Supplementary Figure 2B). The HIF-1α inhibitor also elevated GI_50_ in CD90^−^ cells (Supplementary Figure 2C). These results demonstrated that HIF-1α and its downstream pathways exert growth suppressive functions under conditions of ammonia accumulation. To further elucidate the role of HIF-1α during stress response to ammonia, the percentages of apoptotic and live cell were determined in CD90^+^ PEO1 cells in the presence or absence of shRNA-HIF-1α. Interestingly, the ratio of apoptotic cells increased over time in HIF-1α-KD cells and as a result, the live cell populations diminished while no significant changes were seen in control cells (Figure 3D, 3E). Taken together, these results show that HIF-1α activated under high ammonia levels leads a reduction of cell proliferation but improves cell survival and persistence against ammonia stress.

### Coordinated stress response mediated by HIF-1α and GS against ammonia

**Figure 4:**
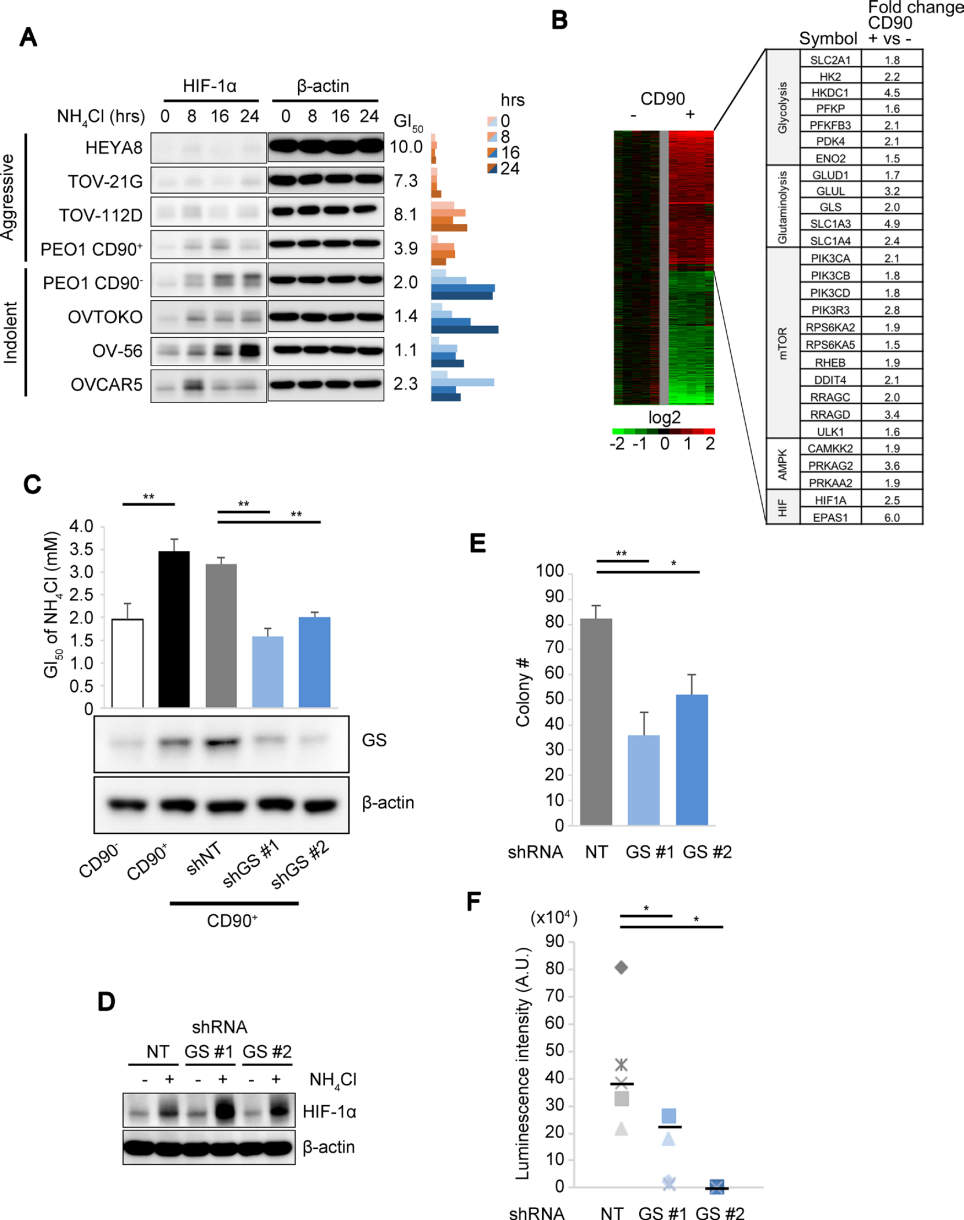
GS drives continued cell proliferation under ammonia stress (**A**) Immunoblot comparison of time-dependent HIF expression levels over time after treatment with 10 mM of NH_4_Cl in aggressive (HEYA8, TOV-21G, TOV-112D and CD90^+^ PEO1) and indolent (CD90^−^ PEO1, OVTOKO, OV-56 and OVCAR5) OVC cell lines. The values of the GI_50_ of NH_4_Cl as determined in Figure 1A are shown in the middle. Right panel bar chart shows the ratio of HIF-1α to β-actin levels loading as determined by immunoblot. Band intensities were measured using Image J. (**B**) Gene expression microarray heatmap showing more than 3,500 genes up- or down-regulated in CD90^+^ cells compared to CD90^−^. Right table shows selected genes up-regulated in the CD90^+^ subpopulation, which are components of the metabolic pathways as indicated. (**C**) The GI_50_ of NH_4_Cl (upper panel) and GS protein expression (lower panel) were determined for the PEO1 CD90^+^/CD90^−^ system and CD90^+^ cells with two independent GS-KD and control knockdown (NT; non-targeting). (**D**) Effect of GS-KD on HIF-1α activation levels upon 10 mM NH_4_Cl treatment. (**E**) Anchorage-independent colony formation was assessed in CD90^+^ PEO1 cells with shNT or GS-KD. (**F**) *In vivo* tumor growth measured using luciferase bioluminescence and IVIS Imaging (*n* = 5 for shNT and shGS #1; *n* = 4 for shGS #2). β-actin was used for loading control in (C and D). Error bars indicate s.e.m. ^*^P < 0.05; ^**^P < 0.01 (Student’s *t*-test).

While HIF-1α activation resulted in attenuated cell growth in CD90^+^ PEO1 cells and promoted cell survival as described above, this could not account for why the cancer stem-like cells presented growth advantages under high ammonia (Figure 1D). Of note, HIF-1α induction levels were limited in 4 OVC cell lines including CD90^+^ PEO1 with more “aggressive” characteristics exhibiting higher NH_4_Cl GI_50_ and increased colony forming efficiencies in soft agar compared to 4 other more “indolent” OVC cell lines with lower GI_50_ and lower colony forming potential including CD90^−^ PEO1 cells (Figure 4A). This led us to hypothesize that the HIF activation by ammonia might be negatively affected by some metabolic factors that were involved in ammonia disposal in the stem-like cells as well as in promoted cell growth of the aggressive OVC cells. We attempted to identify these factors by performing whole transcriptome microarray studies on the PEO1 CD90^+^/CD90^−^ system to determine what were the underlying differences between them that could account for their respective response. There was a preferential up-regulation of genes related to glycolysis and glutaminolysis as well as HIF-1α (*HIF1A*) and HIF-2α (*EPAS1*) expression in CD90^+^ cells (Figure 4B). In particular, we identified ∼3.2 times higher *GLUL* (glutamate-ammonia ligase, GS) mRNA expression and also higher GS protein levels in CD90^+^ PEO1 cells compared to CD90^−^ cells (Figure 4C). Consistently, the decrease in ammonia concentration upon NH_4_Cl load was significantly greater in CD90^+^ cells (Supplementary Figure 3A). GS and its tumor-promoting function have been reported [21, 22] and high GS expressions in OVC tissue and poor patient survival with high GS levels in OVC as well as other cancers were detected (Supplementary Figure 3B–3E). Notably, the NH_4_Cl GI_50_ significantly decreased in line with GS levels in GS-KD cells (Figure 4C). Taken together, these suggest that GS accounts for ammonia disposal and thus promotes cell proliferation in the stem-like cells as it is known to be a rate-limiting enzyme in the condensation reaction of ammonia and glutamate. Interestingly, the activation levels of HIF-1α were increased in 2 independent GS-knockdown (GS-KD) cells (Figure 4D). These results suggest that GS represses HIF-1α protein activation and thus its growth suppressive function. In addition, it is possible that GS itself may be able to promote tumor growth via improvements in glutamine metabolism [21, 22]. Indeed, the colony numbers in soft agar and the tumor growth in the mouse peritoneal cavity were significantly suppressed by GS-KD (Figure 4E, 4F). Collectively, these results suggest a biphasic stress response strategy, in which GS is a predominant factor in ammonia disposal and thus in accelerated proliferation in OVC and CSC-like cells and if ammonia levels exceed the capacity of GS, HIF-1α is activated to attenuate cell growth and promote cell survival.

### HIF-1α channels fuel dependencies in response to ammonia levels

**Figure 5:**
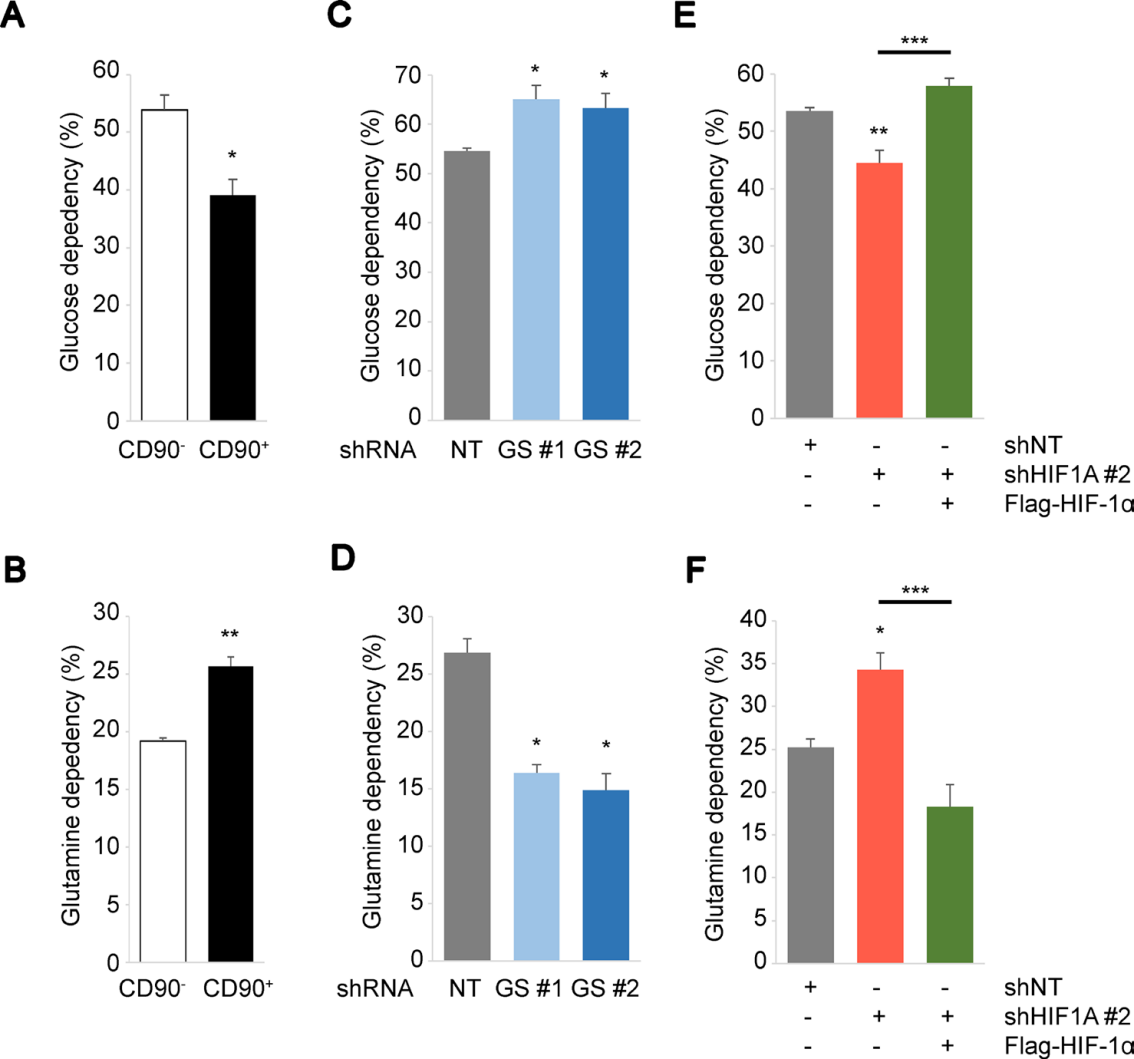
Biphasic regulation of cellular metabolism driven by GS and HIF-1α (**A–F**) Glucose (A, C, E) or glutamine (B, D, F) dependency were determined in CD90^−^ and CD90^+^ PEO1 cells (A, B), in CD90^+^ cells for GS knockdown (C, D) or for HIF-1α knockdown and overexpression over KD (E, F). Glucose/glutamine dependencies were determined using the Seahorse Mito Fuel Flex test. The data show the mean of at least *n* = 3 independent experiments. Error bars indicate s.e.m. ^*^P < 0.05; ^**^P < 0.01; ^***^P < 0.001 (Student’s *t*-test).

We have revealed high GS expression and HIF activation in the stem-like cells, which provide a dynamic stress response mechanism against ammonia. However, the implications of these factors on metabolic regulation under high ammonia are yet to be addressed. We next determined the metabolic fuel dependencies of PEO1 CD90^+^/CD90^−^ cells in the context of GS or HIF-1α depletion. CD90^+^ PEO1 cells had the lower glucose dependencies and the higher glutamine dependencies compared to CD90^−^ cells (Figure 5A, 5B). Interestingly, GS-KD significantly increased the glucose dependencies and decreased the glutamine dependencies (Figure 5C, 5D). These suggest that CD90^+^ PEO1 cells prefer glutamine over glucose compared to CD90^−^ cells and that this dependence was contingent upon GS expression. In contrast, HIF-1α-KD increased glutamine dependency and decreased glucose dependency, which were rescued by Flag-HIF-1α overexpression (Figure 5E, 5F). These results not only confirm that HIF-1α protein functions canonically as a glycolytic regulator, but also suggest its extensive roles in glutamine metabolism. Indeed, HIF-1α has been implicated in the regulation of glutaminolysis in previous studies [29, 30]. Collectively, our studies provide a basis of energy metabolism in response to ammonia stress that is required for fine-tuning of fuel usage and furthermore for cell growth and survival of cancer and CSC-like cells.

## DISCUSSION

**Figure 6:**
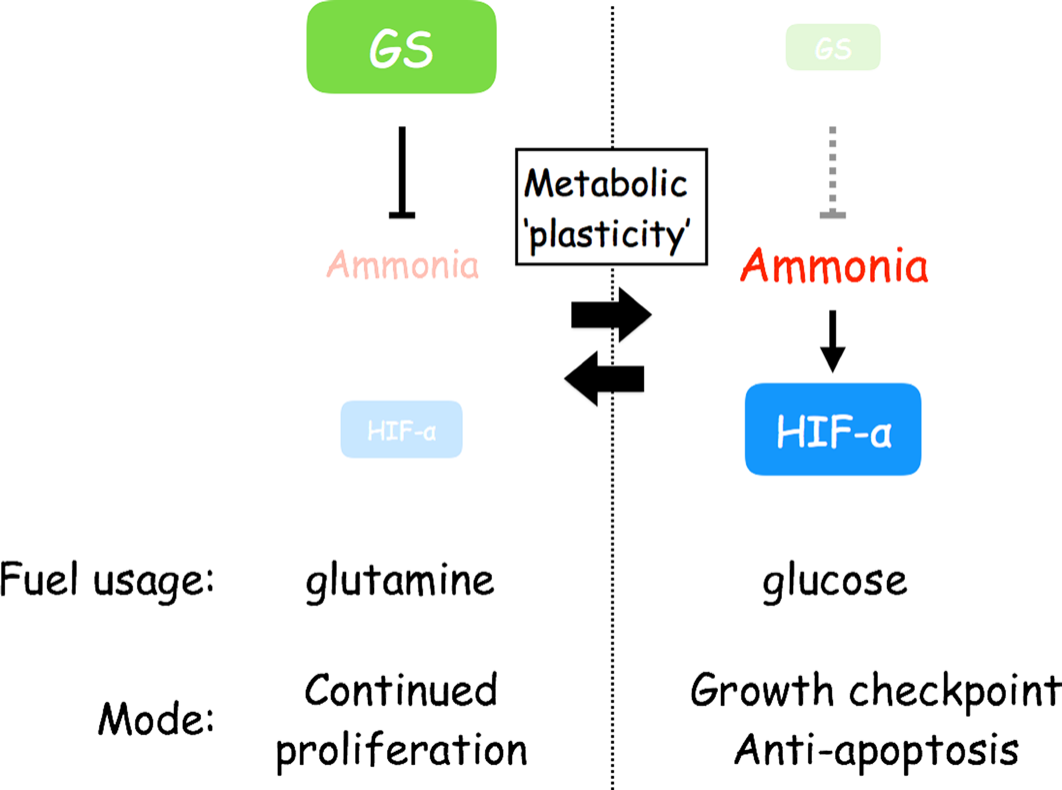
Scheme depicting the flexible stress response against ammonia that is driven by GS and the activation of HIF-1α and relevant for metabolic plasticity

We identified a molecular pathway elicited by ammonia, which activates HIF-1α and regulates cell metabolism, proliferation and survival that may play crucial roles in the tumorigenicity of CSCs. Critically, coupled with the GS function that stimulates glutaminolysis and enhances cell growth we demonstrated here, the HIF-1α activation under high levels of ammonia provides a flexible mechanism of metabolic stress adaptation (Figure 6). These provide a knowledge of metabolic ‘plasticity’ of CSCs, by which they flexibly and dynamically adapt to their internal and/or external microenvironments for cell survival and tumor development [31]. Therefore, our studies may present novel avenues for therapeutic applications.

Although the detrimental effects of ammonia have been well studied [1, 2, 6–10], the implications behind ‘ammonia tolerance’, which is represented by the GI_50_ of NH_4_Cl in this report, for cancer cell growth and the means by which cells tolerate the metabolic stress imposed by ammonia have been unclear. We hypothesize that cancer cells face a dilemma in their development where cells require more glutamine for their growth that leads to the production of ammonia, which in turn suppresses cell proliferation and induce apoptosis. At that rate, this raises the question of what factors offer a solution for continued cell growth and tumorigenicity. To address this, we established the CD90^+^/CD90^−^ PEO1 system as a CSC research platform and found it is a profitable tool for the current study. Although expression of CD90 in CSCs has been increasingly studied, its availability in identifying ovarian cancer stem cell has not been reported. Nonetheless, we demonstrated that CD90-expressing PEO1 cells bear tumor-initiating properties and interestingly, ammonia tolerance (Figure 1B–1D). When cells undergo ammonia stress, glutamine anabolism and thus ammonia condensation is facilitated [32] that would lead to decreased glutamine usage and growing dependence on glucose. Indeed, glucose uptake was elevated by NH_4_Cl treatment (Figure 2D). Our studies demonstrate that activated HIF-1α plays an important role in the metabolic shift (Figure 5A–5D). Glucose-derived fuel for the TCA cycle is shut off by HIF-1α and α-KG supply from glutamine is widely reduced under ammonia stress. In addition, we observed reduced oxygen consumption and ATP levels by NH_4_Cl treatment (Supplementary Figure 4A and 4B). These may support the notion that ammonia causes energy crisis leading to growth defect and/or apoptosis. Our results show that the CSC-like cells as well as aggressive OVC cells had advantages in cell growth and the survival under conditions of high ammonia (Figure 1A, and 1E). 10 mM NH_4_Cl used in the current studies is considered concentrated compared to the levels (5–35 μM) of serum ammonia in healthy individuals while they soar above 1 mM during genetic defects of the urea cycle or liver disease [1]. The previous study showed that the ammonia levels in culture media reach 1.3 to 2.9 mM in 3 days, indicating that ammonia can be accumulated in such closed systems. Coupled with the finding that the levels of ammonia in peripheral tissue are higher than those in the blood (Supplementary Figure 4C), cancer cells deep inside the tumor mass, which would represent cancer stem cell niches [33], could undergo much higher levels of ammonia. In addition, 10 mM NH_4_Cl treatment are most widely used in recent cell biological studies [10, 34]. It is possible especially in the early stages of tumorigenesis that cancer cells are subjected to environmental and cellular stresses and that only cells that tolerate and successfully override these selective pressures may experience expansion to form detectable tumor masses. Our data suggest that the development of mechanisms against ammonia-derived cellular stress is an important requirement for cancer cell aggressiveness (Figure 1A) and furthermore for the survival of the CSC-like cells (Figure 1E).

We identified a HIF protein activation mechanism during the ammonia stress response, which implicates the roles of PHD and HIF in sensing metabolic stress response (Figure 2A, and 2F). It has been well established that the PHD-HIF axis plays central roles in hypoxic sensing and response, in which PHDs are activated by oxygen to hydroxylate and suppress HIFs that are effector transcription factors [11–14]. Besides their functions in the ‘core’ regulatory machinery, accumulating evidence suggests that the PHD-HIF axis is an adaptable system by which cells are capable of sensing changes in metabolism as well as oxygen levels in their microenvironment [35–37]. Our data suggested that HIF is activated by inhibition of prolyl-hydroxylation at least partially mediated by NOS-mediated NO production during ammonia metabolism (Figure 2F, and 2H). The NO production levels upon NH_4_Cl treatment, however, was limited while hydroxylation of HIF-1α (OH-HIF-1α) was almost completely eliminated compared to MG132. This implies other unknown mechanisms behind HIF activation by ammonia. PHD needs metabolic intermediates such as α-KG and bivalent iron for prolyl-hydroxylation of the HIFs [14, 16, 38, 39]. Of note, during the disposal of ammonia by GS, cataplerotic reactions remove α-KG from the TCA cycle [32, 40], which may lead to disturbed TCA cycle and transient accumulations of TCA cycle intermediates that cause HIF-1α stabilization [36]. Thus, we speculate that the extent of the PHD-HIF functions may cover a broad range of changes in cellular metabolism. Interestingly, the HIF-1α expression levels under 1% O_2_ were higher than under NH_4_Cl and they were almost consistent in the presence or absence of NH_4_Cl under hypoxia (Figure 4D). It is suggested that the PHD-HIF axis may play a role in fine-tuning of cellular response during ammonia stress, which could result in divergent consequence compared to hypoxia. Importantly, we demonstrated that HIF-1α inhibits ammonia-induced apoptosis and promotes cell survival while cell proliferation is attenuated (Figure 3A, and 3E). Our study collectively supports the notion that the PHD-HIF axis orchestrates a dynamic cellular response to environmental stresses via regulating the fuel dependency, cellular proliferation and survival and that the role of HIF-1α in stress response against ammonia is not widely divergent from its roles in low oxygen conditions. HIF functions in cellular metabolism and their implications in cancer, however, remain complex. Therefore, further studies are needed to investigate the extent of HIF-mediated transcriptional regulations that contribute towards dynamic stress adaptation.

We report that GS has a pro-oncogenic activity and is highly expressed in the stem-like PEO1 cells as well as aggressive OVCs (Figure 4A, 4C, 4E, and 4F). In agreement with this, recent studies have reported that GS contributes to *de novo* biosynthesis of nucleotides and thus promotes tumor growth [21, 22]. While one study suggested that glioma cells express GS to produce glutamine from glutamate and ammonia, which in turn facilitates purine synthesis from glutamine during glutamine starvation, another team reported that GS is induced and thus nucleotide synthesis is stimulated by oncogenic Myc that is known to up-regulate glutaminolysis, suggesting paradoxical functions of Myc in glutamine metabolism. Likewise, our studies showed that in spite of its enzymatic activity in glutamine synthesis, GS has an important function in maintaining glutamine-dependent metabolism and thus driving the proliferative potential of the cancer stem-like cells (Figures 4E, 4F, 5B and 5D). The importance and implications of glutamine metabolism in cancer cell growth have long been known since the discovery that glutamine is an essential component for the growth of cancer cells in culture media [3]. While the enzymology of GS has been well determined, the dynamics of functional regulation of glutamine metabolism has yet to be elucidated. We hypothesize that in response to changes in ammonia levels in the cellular microenvironment, the regulatory mechanisms mediated by HIF-1α may dictate the direction of glutamine metabolism; glutaminolysis or glutamine synthesis. Indeed, we show that the glutamine dependency of CD90-expressing stem-like cells is inversely regulated by HIF-1α expression levels (Figure 5F). Collectively, our results suggest that the PHD-HIF-1α axis dynamically improves predominance of the energy source in the stem-like cells between glutamine and glucose under ammonia stress, which operates in favor of tumor development although the detailed mechanisms behind this pivot remain to be elucidated. Furthermore, we speculate that by targeting GS therapeutically, cancer cell metabolism may become more dependent on HIF-driven glycolysis with high ammonia levels thereby limiting the metabolic ‘plasticity’, providing the possible means of revealing the vulnerability of CSCs.

## MATERIALS AND METHODS

### Cell culture

A2780 (ECACC, Salisbury, UK), OV56 (ECACC), OVTOKO (JCRB Cell Bank, Ibaraki, Japan), OVISE (JCRB Cell Bank), OV-90 (ATCC, Manassas, VA), PA-1 (ATCC), PEO1 (CRT, Coralville, IA), SKOV3 (ATCC), TOV-112D (ATCC), TOV-21G (ATCC) and UBW1-289 (ATCC) cells were grown in RPMI-1640 media (Nacalai Tesque, Suita, Japan) containing 10% FBS (Serana, Pessin, Germany or Sigma, St. Louis, MO). CaOV-2, HEYA8, OVCAR-5, OVCA433 and OVCAR2 cells were obtained from SGOCL(43), an ovarian cancer cell line library [41] and also cultured in RPMI-1640 media. A498 cells were grown in DMEM media (Nacalai Tesque) containing 10% FBS and 2 mM Glutamax (Thermo Fisher, Waltham, MA). All cell lines used in this study are authenticated by the Centre for Translational Research and Diagnostics (CTRAD, Singapore) or certified by ATCC within 5 years. Mycoplasma test was routinely done during the period of research by using the MycoAlert Mycoplasma Detection Kit (Lonza, Basel, Switzerland). Cells were incubated in a Forma Steri-Cycle CO_2_ incubator (Thermo Fisher) at 37°C with 5% CO_2_ and ambient oxygen level (approximately 20.9% O_2_). Hypoxia treatment was performed in a Invivo2 hypoxia work station (Baker Ruskinn, Sanford, ME, USA) at 37°C under a 5% CO_2_, 1% O_2_ and N_2_-balanced atmosphere. For HIF-1α induction, cells were cultured with 10 mM NH_4_Cl (ammonium chloride, Sigma), 150 μM CoCl_2_ (cobalt(II) chloride hexahydrate, Sigma) or 10 μM MG132 (Z-Leu-Leu-Leu-al, Sigma). To test the involvement of NO or other species of reactive oxygen, 500 μM L-NAME (L-NG-Nitroarginine methyl ester, Sigma) or 25 U/mL SOD (superoxide dismutase, Sigma) was added in the growth media.

### RNAi knockdown and overexpression

For lentivirus productions, 293T cells were co-transfected with shRNA vectors or overexpression vectors with packaging vectors and incubated overnight. The transfection media was replaced with complete growth media (DMEM) and the resulting supernatant was collected 48 hours after changing media followed by filtration using 0.45 μm-pore syringe filter (Sartorius, Göttingen, Germany) and stored at −80°C. For infection, typically, 400, 000 cells were infected with 4 mL of the supernatant with polybrene (8 μg/mL, Sigma) for 4 hours. Puromycin (Sigma) was added at 2 ng/mL 48 hours after infection to establish stable cell lines. The vectors used were pLKO.1-based shRNAs against HIF1A #1 (TRCN0000003811, Sigma), HIF1A #2 (TRCN0000010819, Sigma), GLUL #1 (TRCN0000343992, Sigma) and GLUL #2 (TRCN0000344059, Sigma). To rescue the HIF-1α knockdown, Flag-HIF-1α was cloned into pCSII-CMV-MCS-IRES2-Venus vector (RIKEN, Tsukuba, Japan) and overexpressed using lentivirus system coupled with shRNA HIF1A #2 that targets 3′ UTR region. Human flag-tagged wild-type VHL gene was cloned into pCSII-CMV-MCS-IRES2-Venus vector and overexpressed in A498 cells to establish a stable cell line. For siRNA knockdown, Silencer^®^ Select siRNA-PDK1 (s10252, Thermo Fisher) was transfected with Lipofectamine^®^ RNAiMAX reagent (Thermo Fisher) according to the manufacture’s instruction. Briefly, 10 pmol of the reconstructed siRNA oligo was mixed with 3 μL of the transfection reagent and added onto CD90^+^ PEO1 cells with 100 μL Opti-MEM^®^ I reduced serum media in 12-well plate (Greiner bio-one, Kremsmünster, Austria) followed by 4 hours’ incubation. Cells were allowed to grow for 24 hours and used for subsequent experiments.

### Immunoblotting

Cells were harvested in M-PER reagent (Thermo Fisher) containing complete™ protease inhibitor, PhoSTOP phosphatase inhibitor (Roche, Basel, Switzerland) and PMSF (Sigma). Each 10 μL of denatured samples were separated on Mini-PROTEAN^®^ TGX™ Precast Gels (Bio-Rad, Hercules, CA, USA) and transferred to methanol-activated Hybond P membrane (GE Healthcare, Pittsburgh, PA, USA). Membranes were blocked with TBS-T buffer containing 5% skimmed milk followed by incubation with anti-GLUL (GS) (HPA007316, Sigma), anti-β-actin (A2228, Sigma), anti-α-tubulin (sc-23948, Santa Cruz, Dallas, TX), anti-HIF-1α (GTX127309, GeneTex, Irvine, CA), anti-HIF-2α (7096, Cell Signaling, Danvers, MA, USA), anti-hydroxy-HIF-1α (Pro564, OH-HIF) (3434P, Cell Signaling), anti-GLUT1 (ab15309, Abcam, Cambridge, UK) or anti-VHL (2738, Cell Signaling) antibodies. For multiple protein detection, a membrane was cut into two or three pieces with a good margin. The uncropped images of immunoblots were shown in Supplementary Figures 5 and 6. Bands from multiple blots in a single experiment were normalized by Ponceau-S staining.

### Soft agar assays

Trypsinized cells were resuspended in RPMI-1640 media without phenol red (#R8755, Sigma–Aldrich) containing 0.35% low-melt agarose (#1613111, Bio-Rad) and 10% FBS (Sigma). The cell suspension was plated on top of a 0.5% agarose base layer. The cells were allowed to grow in a Forma Steri-Cycle CO_2_ incubator (Thermo Fisher) CO_2_ incubator at 37°C with 5% CO_2_ for 3 weeks and stained with 0.05% crystal violet (Gentian Violet, ICM Pharma, Singapore). Images of colonies were acquired using a dissecting microscope (SZX-12, Olympus, Tokyo, Japan) and the colony numbers and sizes were quantitated using Image J software (Rasband, W.S., ImageJ, U. S. National Institutes of Health, Bethesda, Maryland, USA, imagej.nih.gov/ij/, 1997–2016).

### Fluorescence-activated cell sorting (FACS)

Cells were trypsinized and resuspended in HBSS containing 2% BSA followed by staining with anti-CD90-PE (#IM1840U, Beckman Coulter, Brea, CA, USA). Sytox-Blue (Thermo Fisher) was used for dead cell staining. Cells were sorted using the BD FACSAria or analyzed using the BD LSR II with the BD FACSDiva Software (BD Biosciences, San Jose, CA, USA).

### Apoptosis assays

To monitor the time-dependent progression of ammonia-induced apoptosis, the duplicate groups of NH_4_Cl-treated cells were allowed to grow in 6-well plates (Greiner Bio-One) with standard culture conditions for 0, 1, 3 or 5 days. To determine the rate of apoptosis, cells were trypsinized and stained with Annexin V, Alexa Fluor^®^ 647 conjugate and SYTOX^®^ Blue nucleic acid stain (Thermo Fisher). The percentage of apoptotic cells (Annexin V-positive and SYTOX blue-negative) and live cells (Annexin V-negative and SYTOX blue-negative) were determined by flow cytometry using the Cell Analyzer EC800 (SONY, Tokyo, Japan).

### Xenograft assays

For serial dilution xenograft studies, the indicated numbers of PEO1 CD90^+^ or CD90^−^ cells were injected subcutaneously into female immunodeficient mice (NOD/MrkBomTac-Prkdc^scid^, InVivos, Singapore) with 200 μl of 50% Matrigel (#354234, BD Biosciences) in HBSS. 8 weeks later, tumors were excised from euthanized mice. CD90^−^ and CD90^+^ PEO1 cells for intraperitoneal injection were infected with pLENTI-luciferase vector (addgene, Cambridge, MA, USA) and selected with puromycin. Luminescence signals from each 1 × 10^6^ cells were tested by adding luciferin substrate and confirmed equivalent. The luciferase-expressing cells were injected into the peritoneal cavities of female NOD/SCID mice. For imaging analysis, RediJect D-Luciferin Bioluminescent Substrate (PerkinElmer, Waltham, MA, USA) was injected into the peritoneal cavity of anesthetized mice at 150 mg/kg, followed by image acquisition using the IVIS Spectrum *In Vivo* Imaging System (PerkinElmer). Upon euthanasia, blood samples were obtained and tumor masses together with intact ovaries were excised. Blood samples were immediately centrifuged at 1,000 g for 10 min at 4°C and supernatants were taken as plasma samples. Tumor samples were homogenized using the Dounce tissue grinder (Sigma) and whole cell extracts were obtained in NP-40 lysis buffer (1% Nonidet P-40, 150 mM NaCl, 50 mM Tris-HCl, pH 7.5). NP-40-insoluble fractions were removed by centrifugation at 15,000 g for 10 min at 4°C. The animal protocol was reviewed and approved by The NUS Institutional Animal Care and Use Committee (reference: 2014-00592 (R14-592) and 045/10(A4)13).

### Metabolite assays

Ammonia or glutamine levels were determined using the Ammonia Colorimetric Assay Kit II (BioVision, Milpitas, CA, USA) according to each of the manufacturer’s protocol. Briefly, culture media or whole cell extracts were deproteinized by centrifugation at 10,000 rpm for 30 min in 10Kd Spin Columns (BioVision). A modified Berthelot assay was carried out and ammonia levels were quantified by colorimetry (OD 670 nm) using the Infinite 200 Pro plate reader (Tecan, Männedorf, Switzerland).

Cellular ATP level was measured by using ApoSENSOR ADP/ATP Ratio Bioluminescence Assay Kit (BioVision) according to the company protocol. Briefly, 3,000 cells were plated in 96-well plate in duplicate 1 day prior to analysis. ATP levels were analyzed as luminescence signal after 5 min incubation with ATP monitoring enzyme and nucleotide releasing buffer at room temperature.

### MTS assays

Cells were plated at initial seeding densities of 1,000/well for NH_4_Cl or 3,000/well for 2DG (2-deoxy-D-glucose, Sigma) treatment in each well of 96-well plates and incubated overnight. The next day, the cells were fed with fresh media with or without inhibitors. The CellTiter 96 AQueous One assay (G3581, Promega, Madison, WI, USA) was performed according to the manufacturer’s instructions for day 6 cells treated with NH_4_Cl or day 4 cells treated with 2DG. Treatments were compared to PBS or DMSO controls at 100% to determine the GI_50_ concentration that inhibits cell growth by 50%. To examine the effect on GI_50_ of NH_4_Cl, 400083 HIF-1 Inhibitor (Millipore, Billerica, MA, USA) was used. For all of the MTS assays, the mean ± s.e.m. of biological triplicates performed in technical duplicates (*n* = 6) and triplicates (*n* = 9) are shown.

### Nitric oxide detection

Each 10,000 cells were plated in 96-well clear bottom black polystyrene microplates and incubated in CO_2_ incubators with indicated levels of NH_4_Cl overnight. Fluorescent readings were acquired using the Tecan plate reader after incubations with HBSS containing DAF-2 DA (Diaminofluorescein-2 diacetate, Goryo chemical) for 30 min. Each triplicated experiment was repeated at 3 times to determine the mean ± s.e.m.

### Extracellular flux analyses

Glucose or glutamine dependency/flexibility was determined using the Seahorse XF Mito Fuel Flex test kit and a Seahorse XFe24 Flux Analyzer (Agilent, Santa Clara, CA, USA) according to the manufacturer’s protocols. Briefly, cells were plated at 70–80% confluency in Seahorse XF24 Cell Culture Microplates (Agilent) in triplicates with XF base medium (Agilent) containing 25 mM glucose (Sigma), 2 mM glutamine (Thermo Fisher) and 1 mM sodium pyruvate (Thermo Fisher). Three replicate oxygen consumption rate (OCR) measurements were acquired at baseline and six measurements were taken after the first injection of UK5099 (2 μM) or BPTES (3 μM) and second injections of etomoxir (4 μM) and BPTES or UK5099 for quantification of glucose or glutamine dependency, respectively. The values for glucose or glutamine dependency/flexibility were calculated from each average OCR values as described in the Seahorse user guide.

### Quantitative RT–PCR

Total RNA was extracted using the RNeasy mini kit (Qiagen, Hilden, Germany) and cDNA was synthesized from 1 μg of total RNA with random hexamers using the RevertAid First Strand cDNA Synthesis Kit (Thermo) according to the manufacturer’s instructions. Quantitative RT–PCR was performed using Kapa Sybr Fast qPCR (Kapa Biosystems, Wilmington, MA, USA) on the 7500 Fast Real-Time PCR System (Applied Biosystems, Foster City, CA, USA). PCR reactions were carried out in biological triplicates and technical triplicates (*n* = 9) and relative expression was calculated using the comparative CT method with 18S ribosomal RNA expression as the reference control. See Supplementary Materials for primer sequences.

### Microarray

For microarray expression profiling, total RNA from *n* = 5 biological replicates of CD90^−^/CD133^−^ and CD90^+^/CD133^−^ subpopulations of PEO1 cells was extracted and purified using the Absolutely RNA Nanoprep Kit (Agilent). Microarray hybridizations were carried out by the cDNA microarray service at the Centre for Translational Research and Diagnostics (CTRAD, Singapore) using GeneChip^®^ Human Genome U133 Plus 2.0 Arrays (Affymetrix, Santa Clara, CA, USA). The raw microarray data was normalized using the cross-correlation method (Chua *et al*., 2006) and differential gene expression was identified based on a minimum fold-change cutoff of at least 1.5 for the average of the CD90^+^/CD133^−^ subpopulation over the average of the CD90^−^/CD133^−^ controls. Normalized gene expression profiles were shown on a heatmap as fold-change for CD90^+^/CD133^−^ PEO1 cells over that of CD90^−^/CD133^−^ cells on a log2 scale. The microarray data was deposited in the NCBI Gene Expression Omnibus (GEO) database with the accession number GSE107251.

### Clinical data analyses

The GS staining data in ovarian cancer patients were obtained from the Human Protein Atlas (www.proteinatlas.org, [42]). KM plotter (kmplot.com, [43]) for ovarian cancer, lung cancer and gastric cancer or REMBRANDT (betastasis.com, [44]) for glioma were used for generating Kaplan–Meier survival curves.

### Statistical analyses

Independent one-sample and two-sample *t*-tests were performed as indicated. One-way ANOVA was used to evaluate differences among multiple groups followed by the Tukey’s test.

## SUPPLEMENTARY MATERIALS FIGURES AND TABLES


