# Supplementary material for: Hypoxia-inducible factor-1α promotes cell survival during ammonia stress response in ovarian cancer stem-like cells
Source: Oncotarget. 2017 Dec 7;8(70):114481–94. doi: 10.18632/oncotarget.23010 (PMC5777708; doi:10.18632/oncotarget.23010)
Supplement: Supplementary file 1 [file oncotarget-08-114481-s001.pdf]

# Hypoxia-inducible factor-1α promotes cell survival during ammonia stress response in ovarian cancer stem-like cells

## SUPPLEMENTARY MATERIALS

Primer sequences

| Target gene | | Sequence |
|---|---|---|
| PFKFB3 | F | GCGTCCCCACAAAAGTGTTC |
| PFKFB3 | R | CCGGACTTTCATGGCTTCCT |
| B2M | F | TGCTCGCGCTACTCTCTCTTT |
| B2M | R | TGTCGGATGGATGAAACCCAGA |

shRNA target sequences

| Target gene | | Sequence | Region |
|---|---|---|---|
| HIF1A | #1 | CCGGCGGCGAAGTAAAGAATCTGAACTCGAGTTCAGATTCTTTACTTCGCCGTTTTT | CDS |
| HIF1A | #2 | CCGGTGCTCTTTGTGGTTGGATCTACTCGAGTAGATCCAACCACAAAGAGCATTTTT | 3' UTR |
| GLUL | #1 | CCGGCACACCTGTAAACGGATAATGCTCGAGCATTATCCGTTTACAGGTGTGTTTTTG | CDS |
| GLUL | #2 | CCGGATAACCACTGCTTCCATTTAACTCGAGTTAAATGGAAGCAGTGGTTATTTTTG | 3' UTR |

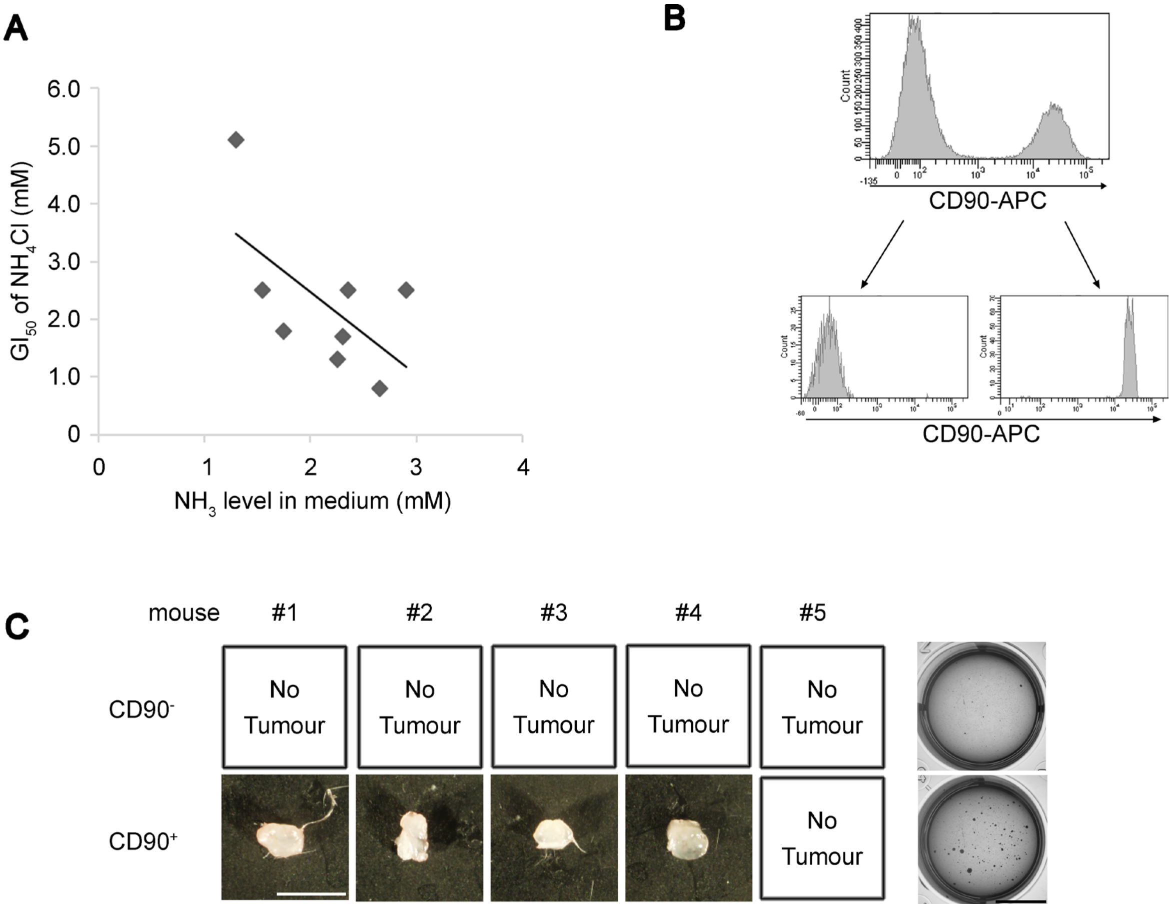


**Supplementary Figure 1:** (**A**) Correlation plot of the $GI_{50}$ of $NH_4Cl$ versus the ammonia levels in culture media. The original data were from Hassell *et al* [6]. (**B**) Outline of cell sorting to establish the PEO1 $CD90^+/CD90^-$ system. APC-conjugated CD90 antibody was used for cell sorting. (**C**) Pictures of xenograft tumors from 5 mice (left panel, scale bar = 5 mm) and representative colony images in soft agar (lower panel, scale bar = 10 mm) of $CD90^-$ and $CD90^+$ PEO1 cells shown in Figure 1B.

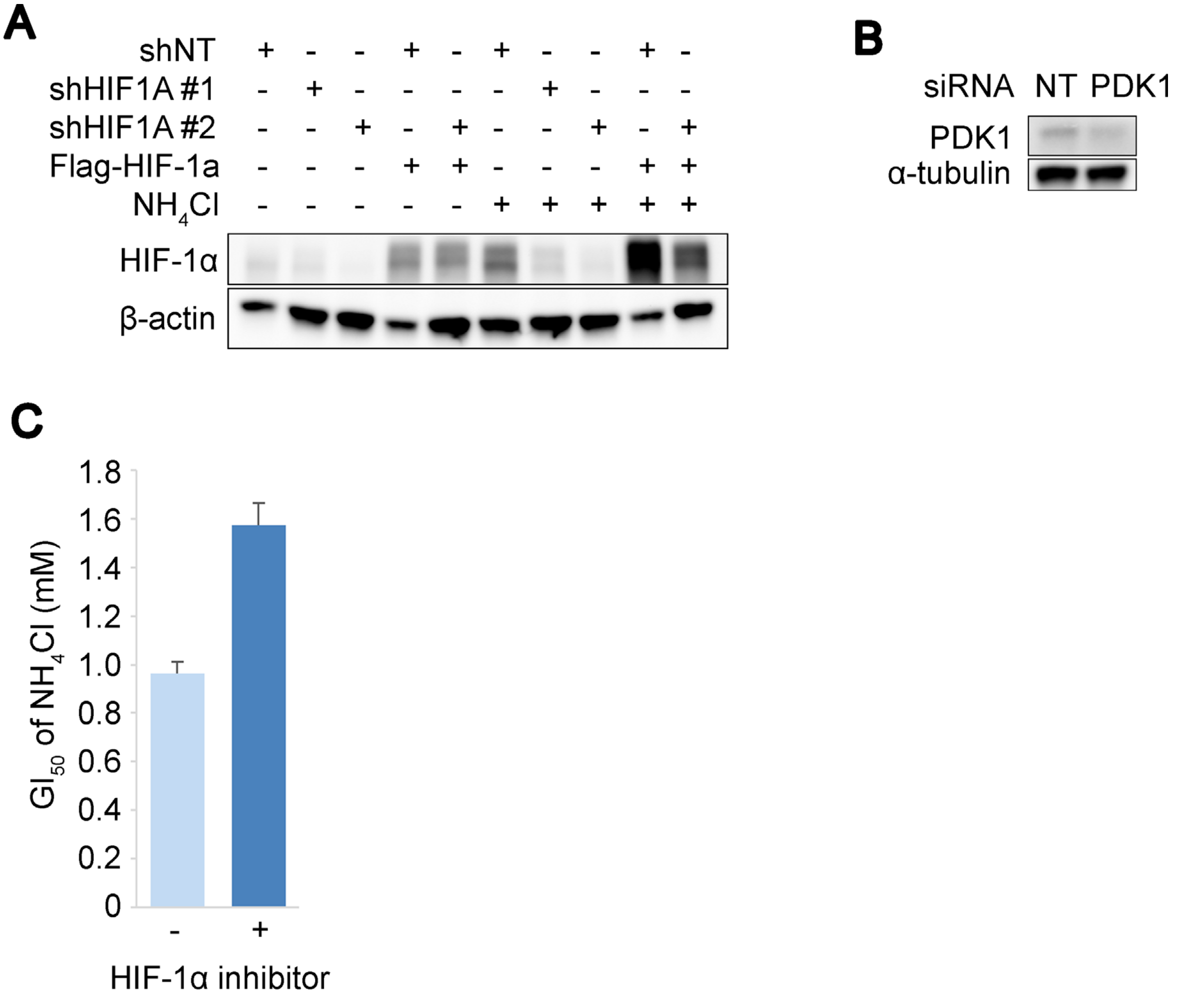


**Supplementary Figure 2:** (**A**) HIF-1α and β-actin expression levels in CD90$^+$ PEO1 cells with two independent *HIF1A* knockdown coupled with Flag-HIF-1α overexpression in the presence or absence of 10 mM $NH_4Cl$ as indicated. (**B**) Validation of PDK1 knockdown. PDK1 and α-tubulin expression levels in the presence or absence of siRNA-PDK1 in CD90$^+$ PEO1 cells. (**C**) Effect of 30 µM HIF-1α inhibitor 400083 on the $GI_{50}$ of $NH_4Cl$ was tested in CD90$^-$ PEO1 cells.

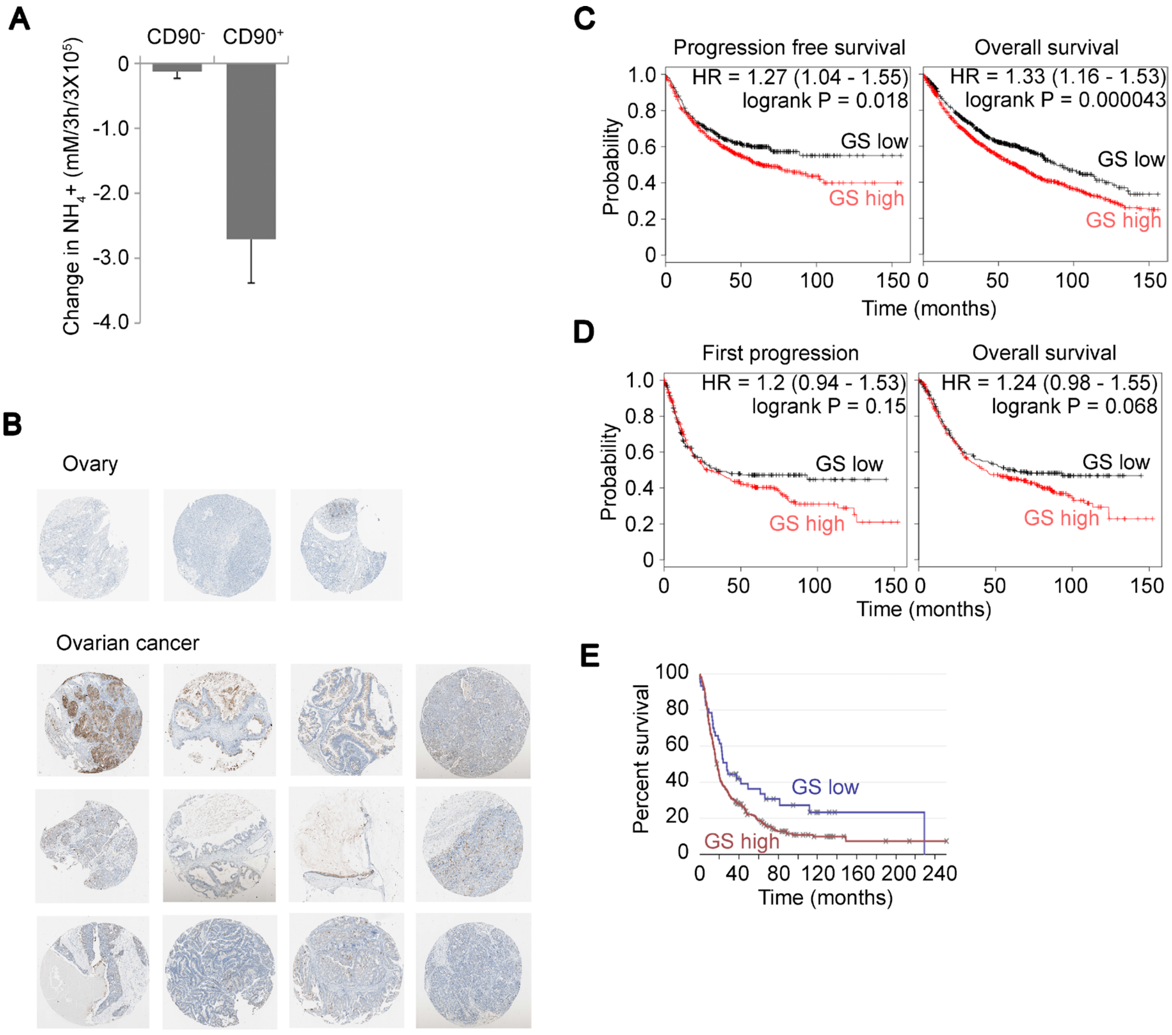


**Supplementary Figure 3:** (**A**) Changes in ammonia levels in CD90$^+$/CD90$^-$ PEO1 cells 3 hours after loading of 10 mM of $NH_4Cl$. The ammonia levels were background subtracted using the cell-free control. (**B**) GS immunohistochemical staining images of normal ovary tissue (upper panel) and OVC tissue (lower panel) from The Human Protein Atlas (www.proteinatlas.org/). Scale bar = 500 µm. (**C**–**E**) Kaplan-Meier plots of *GLUL* (GS) expression in patient tumors with each survival as indicated were generated using KM plotter (C, D) or REMBRANDT (www.betastasis.com/glioma/rembrandt/) (E).

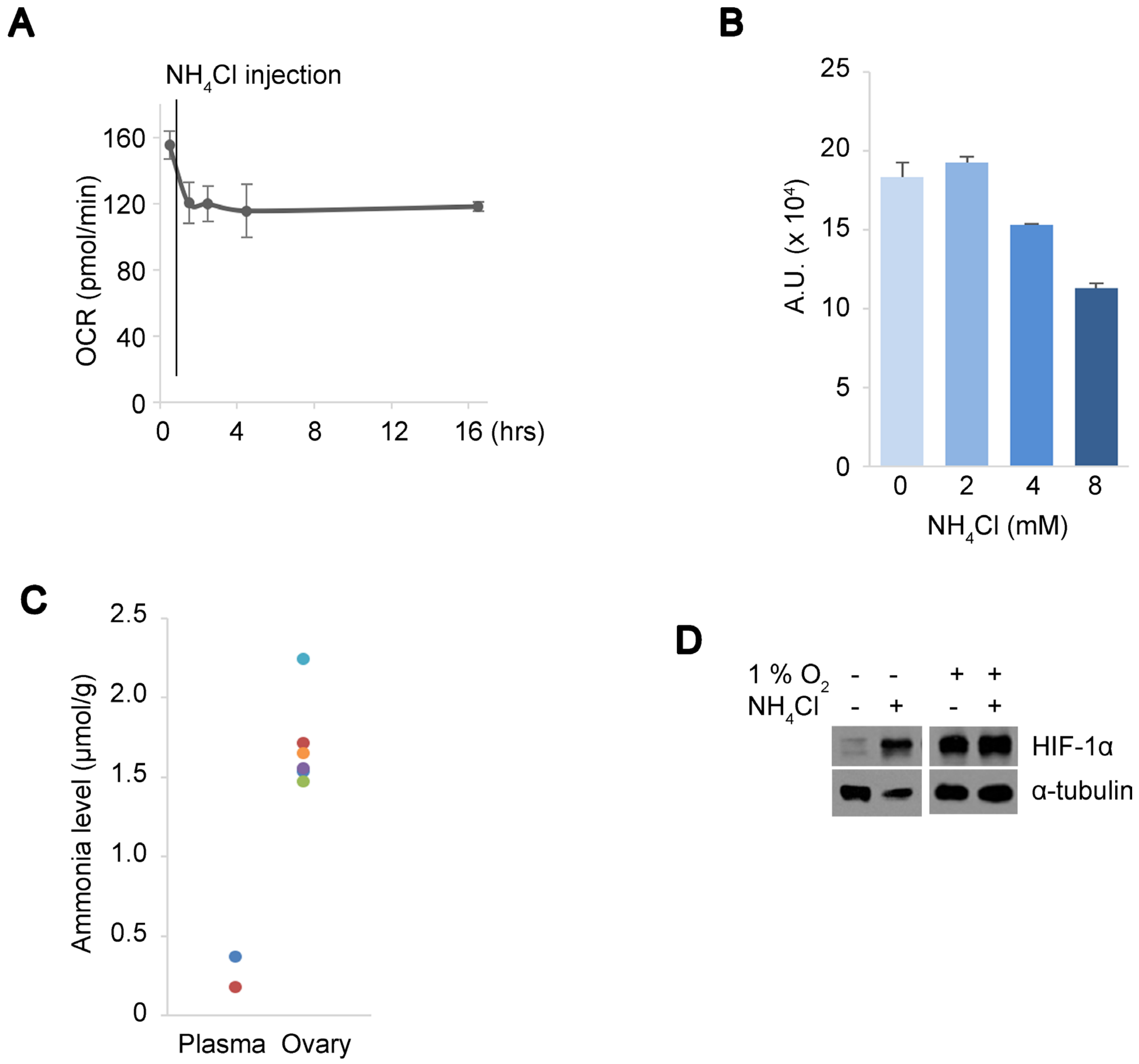


**Supplementary Figure 4:** (**A**) Kinetic oxygen consumption rate (OCR) was analyzed. PEO1 CD90$^{+}$ cells were subjected to 10 mM of $NH_4Cl$ treatment at the 20 min time point followed by monitoring for 16 hrs ($n$ = 3). (**B**) Cellular ATP level was determined. The data shows the mean of a duplicated experiments. Error bars indicate s.e.m. with $^{**}P < 0.01$; $^{***}P < 0.001$ (Student's $t$-test). (**C**) Chart showing ammonia levels in plasma ($n$ = 2) and normal ovary ($n$ = 6). (**D**) HIF-1α protein expression was tested in the presence or absence of 10 mM $NH_4Cl$ under hypoxic (1% $O_2$) or normoxic conditions. α-tubulin shows protein loading.

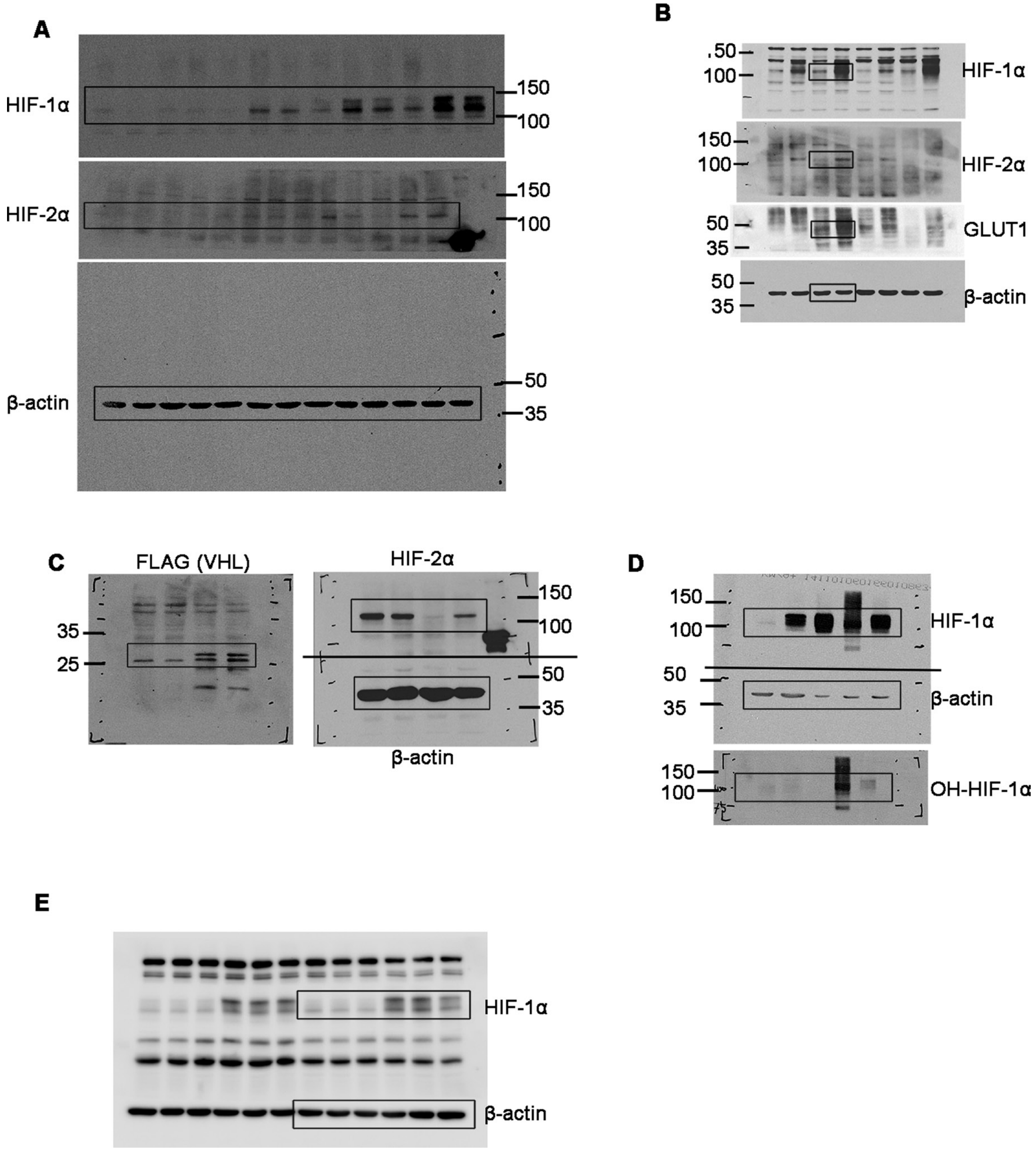


**Supplementary Figure 5: Uncropped images of immunoblots for main figures.** (**A**) HIF-1α (top), HIF-2α (middle) and β-actin (bottom) for Figure 2A. (**B**) HIF-1α, HIF-2α, GLUT1 and α-tubulin (from top to bottom) for Supplementary Figure 2C. (**C**) VHL (left panel), HIF-2α (top right) and β-actin (bottom right) for Figure 2E. (**D**) HIF-1α (top), β-actin (middle) and OH-HIF-1α (bottom) for Figure 2F. (**E**) HIF-1α (top) and β-actin (bottom) for Figure 2H. Black boxes highlight the lanes presented in the Figures. Sliced membranes are separated by empty space or a black line. Each molecular weight is indicated.

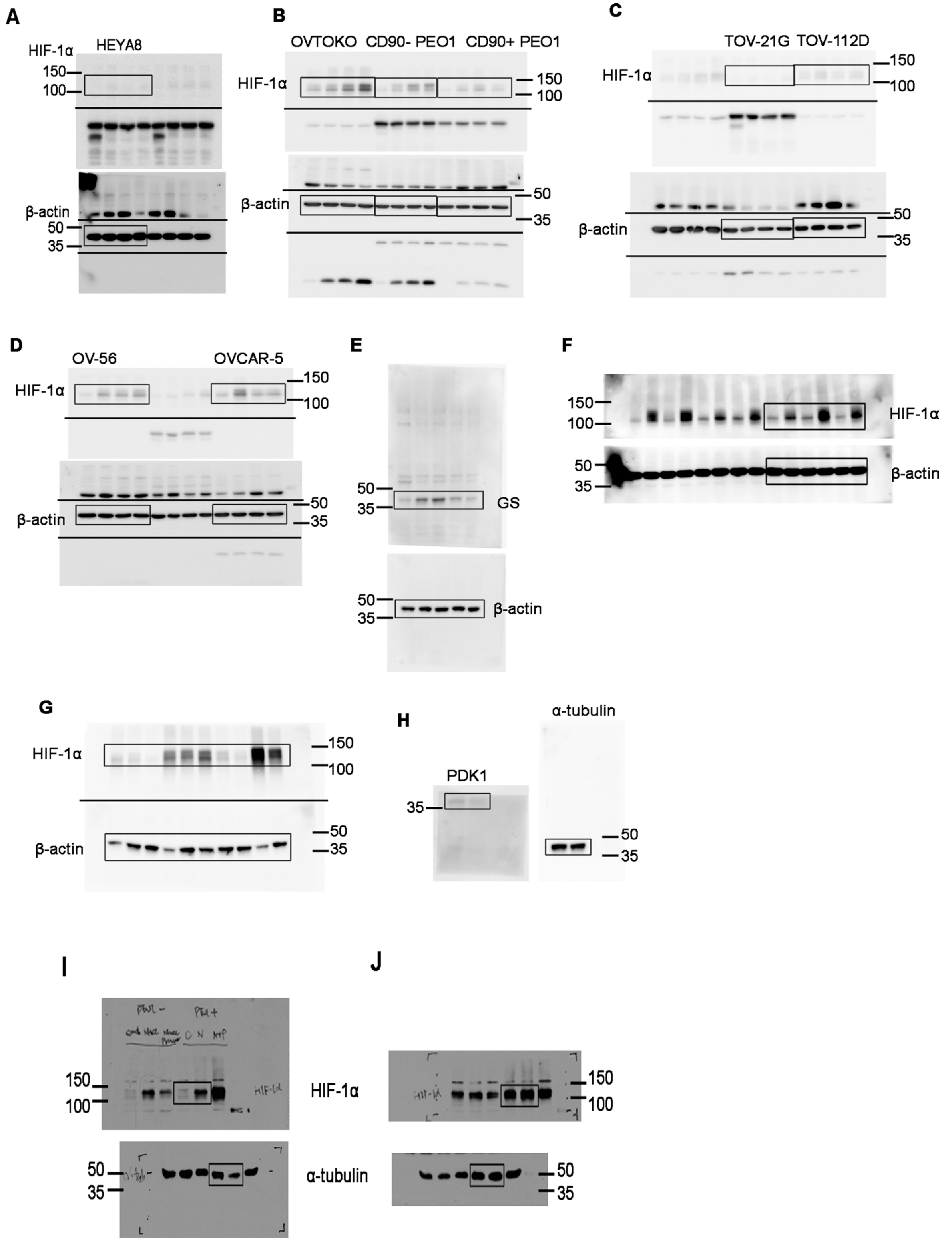


**Supplementary Figure 6: Continued uncropped blot images for main and supplementary figures.** (**A**, **B**, **C**, **D**) Original images for Figure 4A. Top panels show HIF-1α levels and bottom panels β-actin. Each cell line name is indicated (**E**) GS (top panel) and β-actin (bottom) levels for Figure 4C. (**F**) HIF-1α (top panel) and β-actin (bottom) levels for Figure 4D. (**G**) HIF-1α (top panel) and β-actin (bottom) for Supplementary Figure 2A. (**H**) PDK1 (left panel) and α-tubulin (bottom) for Supplementary Figure 2B. (I, J) HIF-1α (top) and α-tubulin (bottom) for Supplementary Figure 4D. Black boxes highlight the lanes presented in the figures. Sliced membranes are separated by empty space or a black line. Each molecular weight is indicated.

**Supplementary Table 1: Summary of the $GI_{50}$ of $NH_4Cl$ and the colony number in soft agar of 15 ovarian cancer cell lines corresponding with Figure 1A**

| # | Cell line | colony # | | | ave | NH4 GI50 | | | ave |
|---|---|---|---|---|---|---|---|---|---|
| 1 | PEO1 | 63.0 | 54.0 | 35.0 | 50.7 | 2.9 | 2.4 | 2.4 | 2.6 |
| 2 | SKOV3 | 114.0 | 87.7 | 111.6 | 104.4 | 5.7 | 5.9 | 10.0 | 7.2 |
| 3 | TOV-21G | 136.5 | 163.0 | 217.0 | 172.2 | 8.5 | 5.1 | 8.3 | 7.3 |
| 4 | TOV-112D | 100.0 | 253.0 | 121.5 | 158.2 | 8.6 | 8.7 | 7.1 | 8.1 |
| 5 | CaOV-2 | 98.5 | 86.6 | - | 92.6 | 10.0 | 10.0 | 10.0 | 10.0 |
| 6 | OVTOKO | 4.0 | 3.3 | 1.0 | 2.8 | 1.5 | 1.6 | 1.1 | 1.4 |
| 7 | OVISE | 0.0 | 0.0 | 0.0 | 0.0 | 1.1 | 2.0 | 1.8 | 1.6 |
| 8 | OVCAR2 | 120.0 | 178.0 | 140.3 | 146.1 | 7.5 | 10.0 | 10.0 | 9.2 |
| 9 | HEYA8 | 90.0 | 43.0 | 80.0 | 71.0 | 10.0 | 10.0 | 10.0 | 10.0 |
| 10 | OV-90 | 47.0 | 31.0 | 42.0 | 40.0 | 5.9 | 5.6 | 6.3 | 5.9 |
| 11 | OV56 | 0.0 | 1.0 | 6.6 | 2.5 | 1.6 | 1.5 | 0.4 | 1.2 |
| 12 | OVCA433 | 0.0 | 0.0 | 0.0 | 0.0 | 1.6 | 2.0 | - | 1.8 |
| 13 | OVCAR5 | 0.0 | 1.0 | 0.7 | 0.6 | 1.4 | 3.0 | 3.2 | 2.5 |
| 14 | A2780 | 28.3 | 27.0 | - | 27.7 | 7.5 | 10.0 | 7.2 | 8.2 |
| 15 | UWB1.289 | 0.0 | 0.0 | 0.0 | 0.0 | 2.9 | 1.7 | - | 2.3 |
